# Case Report: squamous transformation after long-term osimertinib in EGFR-mutant lung adenocarcinoma with retained EGFR/TP53 alterations

**DOI:** 10.3389/fonc.2026.1911851

**Published:** 2026-08-03

**Authors:** Lina Xu, Dongxu Zhang, Xiaoling Chen, Linlin Wang, Qiang Liu

**Affiliations:** 1 Oncology Department of Integrated Traditional Chinese and Western Medicine, Shenyang Tenth People’s Hospital, Shenyang, Liaoning, China; 2 Department of Radiology, Shenyang Tenth People’s Hospital, Shenyang, Liaoning, China; 3 Department of Pathology, Shenyang Tenth People’s Hospital, Shenyang, Liaoning, China; 4 Department of Thoracic Surgery, Shenyang Tenth People’s Hospital, Shenyang, Liaoning, China

**Keywords:** acquired resistance, case report, EGFR mutation, lung adenocarcinoma, osimertinib, re-biopsy, squamous transformation

## Abstract

Histological transformation is an important mechanism of acquired resistance to epidermal growth factor receptor tyrosine kinase inhibitors (EGFR-TKIs) in EGFR-mutant non-small cell lung cancer. Compared with small cell transformation, acquired squamous transformation is uncommon and remains less well characterized. We report a 52-year-old woman who underwent thoracoscopic left upper lobectomy and wedge resection of the left lower lobe for lung adenocarcinoma in April 2021. Molecular testing identified an EGFR exon 19 deletion and a TP53 c.713G>T (p.C238F) mutation. She received osimertinib, and the interval from treatment initiation to the first clinically and radiologically suspected disease progression was approximately 39 months. After this clinically suspected progression, pemetrexed plus cisplatin achieved a partial response. In August 2025, an enlarged left supraclavicular lymph node was detected, and re-biopsy confirmed squamous cell carcinoma. Systemic evaluation did not reveal evidence of a second primary malignancy. Molecular testing of the squamous lesion identified the same EGFR exon 19 deletion and TP53 mutation as those in the primary lung adenocarcinoma, providing molecular evidence consistent with a shared tumor origin and favoring acquired squamous histological transformation over a second primary squamous cell carcinoma. The patient subsequently received carboplatin, nanoparticle albumin-bound paclitaxel, and tislelizumab, achieving a radiologic partial response followed by stable disease during maintenance tislelizumab. This case highlights acquired squamous histological transformation as a rare resistance pattern after long-term osimertinib treatment. Re-biopsy and molecular comparison are important for distinguishing histological transformation from a second primary tumor. The observed response to platinum-taxane chemotherapy combined with tislelizumab should be interpreted cautiously because the relative contributions of chemotherapy and immunotherapy cannot be determined from a single case.

## 1 Introduction

Lung adenocarcinoma is a major histological subtype of non-small cell lung cancer (NSCLC), and epidermal growth factor receptor (EGFR) mutations are frequently detected in Asian patients with lung adenocarcinoma (1). The development of EGFR tyrosine kinase inhibitors (EGFR-TKIs), particularly the third-generation EGFR-TKI osimertinib, has substantially improved outcomes in patients with EGFR-mutant advanced lung adenocarcinoma (2). However, most patients eventually develop acquired resistance. The underlying mechanisms are heterogeneous and include secondary EGFR alterations, bypass signaling activation, downstream pathway changes, phenotypic adaptation, and histological transformation (3).

Histological transformation is increasingly recognized as a clinically important mechanism of resistance to EGFR-TKI therapy (4). Transformation from lung adenocarcinoma to small cell lung cancer has been more frequently reported, whereas transformation to squamous cell carcinoma is relatively uncommon (5, 6). Owing to its rarity, the molecular basis, clinicopathological characteristics, and optimal therapeutic strategy for acquired squamous transformation remain poorly defined (7, 8). In clinical practice, this resistance pattern may be difficult to distinguish from a second primary squamous cell carcinoma, particularly when progression occurs at an extrapulmonary or nodal site.

Histological transformation reflects a change in tumor lineage identity and may have direct implications for subsequent treatment decisions (9). Therefore, re-biopsy is important in patients who develop disease progression during or after EGFR-TKI treatment. Tissue reassessment can identify histological evolution, clarify the mechanism of acquired resistance, and provide material for molecular comparison between the primary tumor and the resistant lesion. This distinction is clinically relevant because a transformed tumor may retain the founder EGFR mutation while showing altered morphology, immunophenotype, and therapeutic behavior.

The optimal treatment strategy after squamous transformation remains unclear. Patients with EGFR-mutant lung adenocarcinoma generally derive limited benefit from immune checkpoint inhibitor monotherapy. Whether histological transformation alters treatment sensitivity, including responsiveness to cytotoxic chemotherapy or chemotherapy-based combination strategies, remains uncertain (10). Herein, we report a rare case of EGFR exon 19 deletion-positive lung adenocarcinoma that developed acquired squamous transformation in a left supraclavicular lymph node after long-term osimertinib therapy. The squamous lesion retained the original EGFR exon 19 deletion and TP53 mutation, findings that were consistent with a shared tumor origin and favored acquired histological transformation over a second primary squamous cell carcinoma. This case highlights the diagnostic value of re-biopsy and molecular comparison while emphasizing the therapeutic uncertainty after squamous transformation.

## 2 Case description

**Figure 1:**
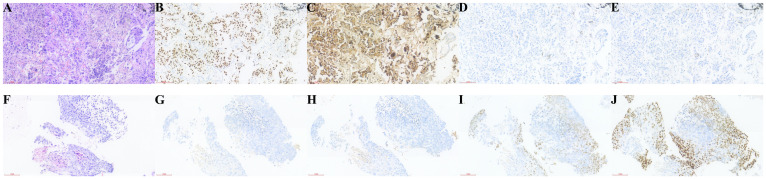
Histopathological and immunohistochemical comparison of the original left upper-lobe invasive adenocarcinoma and transformed squamous cell carcinoma. **(A–E)** Original left upper-lobe invasive adenocarcinoma resected in 2021. **(A)** Hematoxylin and eosin (H&E) staining showing adenocarcinoma morphology. Immunohistochemistry shows **(B)** positive nuclear staining for TTF-1, **(C)** positive granular cytoplasmic staining for Napsin A, **(D)** negative staining for p40, and **(E)** negative staining for p63. No morphological or immunophenotypic evidence of a squamous component was identified. **(F–J)** Squamous cell carcinoma in a left supraclavicular lymph-node biopsy obtained in 2025. **(F)** H&E staining showing squamous cell carcinoma morphology. Immunohistochemistry shows **(G)** negative staining for TTF-1, **(H)** negative staining for Napsin A, **(I)** positive nuclear staining for p40, and **(J)** positive nuclear staining for p63. Original magnification, ×200; scale bars, 100 μm.

A 52-year-old woman underwent thoracoscopic left upper lobectomy, systematic lymph node dissection, and wedge resection of the left lower lobe under general anesthesia in April 2021. Postoperative pathological examination revealed invasive adenocarcinoma in the left upper lobe and a separate minimally invasive adenocarcinoma in the left lower lobe, with pleural metastatic nodules and metastatic involvement of the station 6 and station 13 lymph nodes. Retrospective pathological review and additional immunohistochemical staining showed that the left upper-lobe invasive adenocarcinoma was positive for TTF-1 and Napsin A and negative for p40 and p63, with no identifiable squamous component (**Figures 1A–E**). The metastatic deposits in the station 6 and station 13 lymph nodes were positive for TTF-1 and Napsin A and negative for p40, supporting metastatic pulmonary adenocarcinoma (**Supplementary Figures 1A–H, L, M**). Representative sections of a pleural nodule showed adenocarcinoma morphology with positive staining for TTF-1 and Napsin A (**Supplementary Figures 1I–K**). Based on the concordant histological and immunophenotypic findings, together with the classification of the left lower-lobe lesion as a separate minimally invasive adenocarcinoma, the pleural and lymph-node metastases were attributed to the left upper-lobe invasive adenocarcinoma on integrated clinicopathological grounds. The disease was therefore classified as pT2N2M1a, stage IVA according to the eighth edition of the tumor–node–metastasis classification (11). The primary antibodies used were TTF-1 (clone SP141; Ventana Medical Systems, Inc.), Napsin A (clone MRQ-60; Cell Marque Corporation), p40 (clone BC28; Ventana Medical Systems, Inc.), p63 (clone 4A4; Ventana Medical Systems, Inc.), pan-cytokeratin (AE1/AE3/PCK26 antibody cocktail; Ventana Medical Systems, Inc.), and Ki-67 (clone 30-9; Ventana Medical Systems, Inc.). Immunohistochemical staining was performed on formalin-fixed, paraffin-embedded tissue sections according to the manufacturers’ instructions.

Baseline molecular testing was performed using tissue from the resected left upper-lobe invasive adenocarcinoma. The left lower-lobe minimally invasive adenocarcinoma, pleural nodules, and lymph-node specimens were not tested. The analysis used a tissue-based targeted next-generation sequencing panel covering 31 genes relevant to solid tumors. The assay identified an EGFR exon 19 deletion (c.2236_2250del, p.E746_A750del) and a TP53 c.713G>T (p.C238F) mutation, with variant allele frequencies of 67.76% and 43.42%, respectively. The estimated EGFR copy number was 11.95. No reportable alterations were detected in ALK, ROS1, BRAF, MET, RET, ERBB2, KRAS, NTRK1, or NTRK3 within the tested range.

Although surgery was initially performed with curative intent, postoperative pathological examination revealed pleural metastatic nodules, resulting in postoperative upstaging to pT2N2M1a, stage IVA disease. Accordingly, osimertinib was administered as first-line systemic targeted therapy for EGFR-mutant advanced NSCLC rather than as conventional adjuvant therapy.

In May 2021, the patient began osimertinib 80 mg once daily. In August 2024, the serum carcinoembryonic antigen level increased to 16.18 ng/mL, and chest computed tomography showed increased left pleural effusion. Given the previously documented pleural metastases, these findings were considered clinically and radiologically suggestive of disease progression. Pleural fluid cytology, cell-block examination, and pleural biopsy were not performed at that time; therefore, pleural progression was not pathologically confirmed. From August to November 2024, the patient received four 21-day cycles of pemetrexed (500 mg/m² on day 1) and cisplatin (75 mg/m² per cycle, divided over days 1–3), achieving a partial response. Osimertinib was continued during and after chemotherapy and was permanently discontinued after squamous histological transformation was confirmed in September 2025. Thus, the interval from osimertinib initiation to the first clinically and radiologically suspected progression was approximately 39 months, whereas the total duration of osimertinib treatment was approximately 52 months. Continuation beyond the initial suspected progression was an individualized decision intended to maintain suppression of the known EGFR-mutant adenocarcinoma component until histological transformation was confirmed.

**Figure 2:**
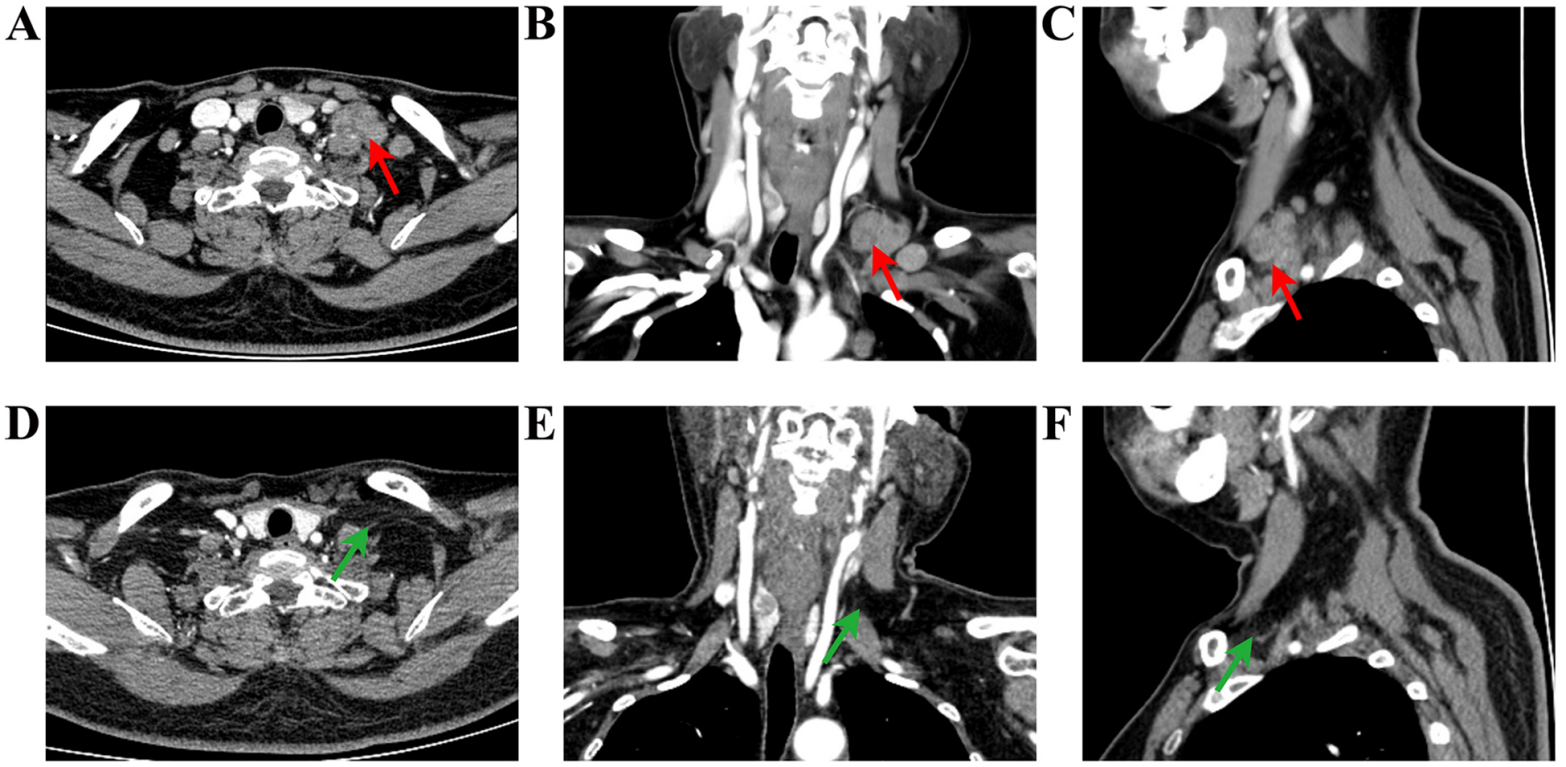
Radiologic changes in the left supraclavicular target lymph node before and after treatment. **(A–C)** Pretreatment contrast-enhanced CT images showing the left supraclavicular target lymph node measuring 22 × 18 mm, with a short-axis diameter of 18 mm. **(D–F)** Follow-up CT images obtained after four cycles of carboplatin, nab-paclitaxel, and tislelizumab showing that the lymph node had decreased to 22 × 7 mm, with a short-axis diameter of 7 mm, representing a 61.1% reduction in the short-axis diameter from baseline.

In August 2025, contrast-enhanced CT of the neck revealed an enlarged left supraclavicular lymph node measuring 22 × 18 mm, with a short-axis diameter of 18 mm. This node was selected as the target lesion according to RECIST version 1.1 (**Figures 2A–C**). In September 2025, ultrasound-guided needle biopsy of the left supraclavicular lymph node was performed. Histopathological examination showed squamous cell carcinoma (**Figure 1F**). Immunohistochemistry showed negative staining for TTF-1 and Napsin A and positive nuclear staining for p40 and p63 (**Figures 1G–J**). Pan-cytokeratin (CKpan) was positive, and the Ki-67 proliferation index was approximately 70% (stains not shown). CK5/6 staining was not performed because of limited biopsy material. Collectively, these findings supported squamous differentiation rather than persistent adenocarcinoma morphology.

To evaluate the possibility of a second primary squamous cell carcinoma, systemic evaluation was performed. Contrast-enhanced brain magnetic resonance imaging showed no intracranial metastasis. Positron emission tomography-computed tomography did not reveal any hypermetabolic lesion suggestive of a second primary tumor in the head and neck region, oral cavity, nasopharynx, upper aerodigestive tract, esophagus, stomach, colorectum, or other organs. Nasopharyngoscopy, gastroscopy, and colonoscopy did not identify an independent primary malignancy. In the context of the patient’s history of EGFR-mutant lung adenocarcinoma, the absence of another primary malignancy on systemic evaluation and the shared EGFR and TP53 alterations favored acquired squamous histological transformation over a second primary squamous cell carcinoma.

From September to December 2025, the patient received four 21-day cycles of carboplatin (AUC 5 on day 1), nab-paclitaxel (200 mg/m² on day 1), and tislelizumab (200 mg on day 1). PD-L1 testing had not been performed before treatment initiation and therefore did not inform the initial treatment decision. According to CTCAE version 5.0, the patient developed a grade 1 white blood cell count decrease after the first cycle, with a nadir of 3.41 × 10^9^/L. She also developed grade 1 peripheral sensory neuropathy, characterized by mild numbness of the hands and feet without limitation of daily activities. The patient received a single subcutaneous dose of recombinant human granulocyte colony-stimulating factor. From the second cycle onward, nab-paclitaxel was administered at 100 mg/m² on days 1 and 8.

Follow-up neck and chest CT in January 2026 showed that the left supraclavicular target lymph node had decreased from 22 × 18 mm to 22 × 7 mm. Its short-axis diameter decreased from 18 mm to 7 mm, corresponding to a 61.1% reduction. The mediastinal lymph nodes also decreased in size, and no new lesions were identified. The overall response was classified as a partial response according to RECIST version 1.1 (12) (**Figures 2D–F**).

**Figure 3:**
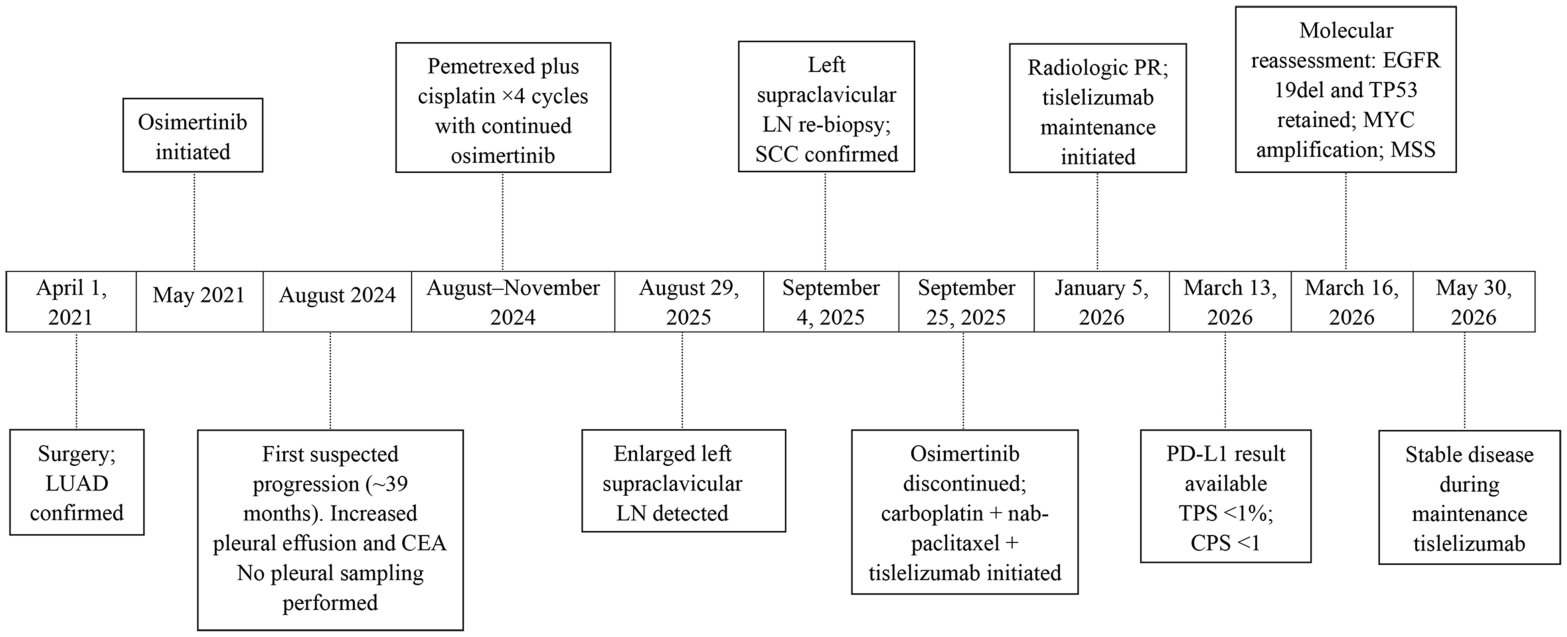
Clinical course and treatment timeline of the patient. Surgery was performed on April 1, 2021, and postoperative pathological examination confirmed lung adenocarcinoma. Osimertinib was initiated in May 2021. Clinically and radiologically suspected disease progression, manifested by increased left pleural effusion and an elevated serum carcinoembryonic antigen level, was identified in August 2024. Neither pleural fluid cytology nor pleural biopsy was performed at that time. The patient then received four cycles of pemetrexed plus cisplatin from August to November 2024, with concurrent continuation of osimertinib. An enlarged left supraclavicular lymph node was detected on August 29, 2025. Re-biopsy of the lymph node on September 4, 2025, confirmed squamous cell carcinoma. Osimertinib was discontinued, and carboplatin, nab-paclitaxel, and tislelizumab were initiated on September 25, 2025. A radiologic partial response was documented on January 5, 2026, according to RECIST version 1.1, after which tislelizumab maintenance was initiated. PD-L1 immunohistochemistry performed on March 13, 2026, using archived tissue from the September 2025 biopsy showed negative PD-L1 expression, with a TPS of <1% and a CPS of <1. Molecular reassessment of the same archived tissue on March 16, 2026, confirmed retention of the EGFR exon 19 deletion and TP53 mutation, detected MYC amplification, and showed that the tumor was microsatellite stable. At the latest follow-up on May 30, 2026, the patient had stable disease during maintenance tislelizumab. Abbreviations: CEA, carcinoembryonic antigen; CPS, combined positive score; EGFR, epidermal growth factor receptor; LN, lymph node; LUAD, lung adenocarcinoma; MSS, microsatellite stable; PD-L1, programmed death-ligand 1; PR, partial response; SCC, squamous cell carcinoma; TPS, tumor proportion score.

Maintenance tislelizumab was initiated at 200 mg every 4 weeks in January 2026 after the patient achieved a partial response with acceptable treatment tolerability. The PD-L1 result was not yet available and therefore did not influence this decision. When the subsequent PD-L1 result proved negative, maintenance treatment was continued because of ongoing disease control and tolerability rather than biomarker positivity. At the latest follow-up on May 30, 2026, the patient had stable disease during maintenance tislelizumab. No immune-related adverse events or other clinically relevant cumulative platinum- or taxane-related toxicities were documented during the reported follow-up period. The clinical course is summarized in **Figure 3**.

In March 2026, archived formalin-fixed, paraffin-embedded tissue from the left supraclavicular lymph-node biopsy obtained in September 2025 was analyzed; no new specimen was obtained. Genetron Health (Beijing, China) performed the analysis using a tissue-based targeted next-generation sequencing panel covering 78 genes. The pathological tumor-cell content was estimated at 35%. The transformed lesion harbored the same EGFR exon 19 deletion (c.2236_2250del, p.E746_A750del) and TP53 c.713G>T (p.C238F) mutation as those in the primary lung adenocarcinoma, with variant allele frequencies of 20.10% and 64.09%, respectively. Because the primary and squamous specimens were analyzed using different targeted sequencing panels and may also have differed in tumor-cell content and other pre-analytical characteristics, their variant allele frequencies were not directly comparable. The presence of the same EGFR exon 19 deletion and TP53 mutation in both specimens supported a common tumor origin and favored acquired squamous histological transformation over a second primary squamous cell carcinoma. This molecular concordance did not, however, definitively establish clonal evolution or clarify the evolutionary relationship between the two lesions. MYC amplification was also detected in the transformed lesion, with an estimated copy number of 5.70. No alterations were detected in MET, PIK3CA, PTEN, or RB1, and no other immediately actionable resistance alterations were identified within the panel’s coverage. The tumor was microsatellite stable. PD-L1 immunohistochemistry using clone E1L3N (Cell Signaling Technology) was negative, with a tumor proportion score (TPS) of <1% and a combined positive score (CPS) of <1.

## 3 Discussion

Acquired resistance remains a major challenge in EGFR-mutant lung adenocarcinoma treated with EGFR-TKIs (13). Reported mechanisms include secondary EGFR alterations, bypass pathway activation, downstream pathway alterations, epithelial-to-mesenchymal transition, and histological transformation (14). Compared with small cell transformation, squamous histological transformation is uncommon, and the available evidence is derived mainly from case reports and small series (15, 16). **Table 1** provides a non-systematic selection of representative reports and studies (5, 8, 15–22). In many of these reports, the original EGFR alteration was retained in the squamous lesion, whereas additional molecular alterations and clinical outcomes varied considerably.

**Table 1:** Selected representative reports and studies of squamous histological transformation in EGFR-mutant lung adenocarcinoma after EGFR-TKI therapy.

Author/year	Cases	Initial EGFR mutation	EGFR-TKI before transformation	Transformation site	Molecular/pathological findings after transformation	Post-transformation treatment and outcome	Key relevance
Ye et al., 2022 (5)	1	L858R	Gefitinib	Local metastatic lung lesions	Retained EGFR L858R; MET amplification; high PD-L1 expression	Paclitaxel, carboplatin and pembrolizumab; disease control for 8 months	Suggests that chemoimmunotherapy may be active in selected PD-L1-high transformed cases
Xi et al., 2022 (8)	1	Exon 19 deletion	Icotinib followed by osimertinib	New lung lesion	Retained EGFR exon 19 deletion and T790M; MET amplification	Osimertinib-based combination therapy; later targeted and immunotherapy regimens; died approximately 12 months after SCC confirmation	Highlights repeat biopsy and molecular reassessment in transformation with concurrent resistance alterations
Park et al., 2019 (17)	4	L858R or exon 19 deletion	Gefitinib, afatinib, erlotinib, or osimertinib	Lymph node, pleura, skin/scalp, or bone/soft tissue	Retained initial EGFR mutation in all transformed SCC samples; PI3K/AKT/mTOR pathway alterations in selected cases	Treatment varied; selected cases received everolimus or osimertinib	Supports a shared tumor origin and possible adenocarcinoma-to-SCC lineage transition
Schoenfeld et al., 2020 (18)	5 SCC transformations among 62 paired cases	Various EGFR mutations	First-line or later-line osimertinib	Resistant tumor tissue after progression	Retained sensitizing EGFR mutation when evaluable; additional off-target alterations in selected cases	Subsequent treatment varied; outcomes were mixed	Identifies squamous transformation as an off-target resistance mechanism to osimertinib and supports tissue re-biopsy
Roca et al., 2019 (19)	17	Exon 19 deletion or L858R in most cases	Gefitinib, erlotinib, afatinib, or sequential EGFR-TKIs	Progressive SCC lesions at re-biopsy	Baseline EGFR mutation retained in transformed SCC lesions; acquired T790M in some cases	Chemotherapy, EGFR-TKI, or combined approaches; median OS after SCC onset was 3.5 months	Shows poor prognosis and limited efficacy of available therapies after SCC transformation
Qiu et al., 2025 (22)	1	Exon 19 deletion	Gefitinib followed by osimertinib	Left clavicular/subclavicular mass	EGFR driver mutation not detected in transformed SCC; p40/p63 positive and TTF-1/Napsin A negative	Carboplatin/docetaxel followed by tislelizumab plus carboplatin/docetaxel; extracranial PR but brain progression; OS after SCC transformation was 8 months	Demonstrates EGFR-positive adenocarcinoma transforming into EGFR-negative SCC with heterogeneous treatment response
Present case	1	Exon 19 deletion; TP53 c.713G>T (p.C238F)	Osimertinib	Left supraclavicular lymph node	Retained EGFR exon 19 deletion and TP53 mutation; MYC amplification; MSS; no MET amplification; PD-L1 TPS <1% and CPS <1	Carboplatin plus nab-paclitaxel and tislelizumab followed by tislelizumab maintenance; PR followed by stable disease	Supports acquired squamous transformation after long-term osimertinib; treatment response should be interpreted cautiously

This table presents a non-systematic selection of representative publications and is not intended to be exhaustive. CPS, combined positive score; EGFR, epidermal growth factor receptor; EGFR-TKI, epidermal growth factor receptor tyrosine kinase inhibitor; MSS, microsatellite stable; nab-paclitaxel, nanoparticle albumin-bound paclitaxel; OS, overall survival; PD-L1, programmed death-ligand 1; PR, partial response; SCC, squamous cell carcinoma; TKI, tyrosine kinase inhibitor; TPS, tumor proportion score.

In this case, the morphology and immunophenotype of the left supraclavicular lymph-node lesion, the absence of another primary malignancy on systemic evaluation, and the shared EGFR exon 19 deletion and TP53 mutation supported a common tumor origin and favored acquired squamous histological transformation. Nevertheless, targeted panel sequencing alone could not establish clonal evolution or define the precise evolutionary relationship between the two lesions. Proposed mechanisms of squamous transformation include intratumoral heterogeneity, treatment-driven clonal selection, transdifferentiation, and lineage plasticity (23, 24). Retention of an EGFR-sensitive mutation may help establish tumor origin but does not necessarily imply continued EGFR dependence after lineage transformation. The biological significance of MYC amplification remains uncertain, and whether it was acquired could not be determined because the paired specimens were analyzed using different targeted panels. Moreover, because pemetrexed plus cisplatin was administered before the transformation was histologically confirmed, a contribution from chemotherapy-related selective pressure cannot be excluded. The transformation therefore cannot be attributed solely to osimertinib.

Repeat tissue biopsy is particularly important when progression during EGFR-TKI treatment follows an atypical clinical or anatomical pattern (25–27). No pleural specimen was obtained when the left pleural effusion increased in August 2024; therefore, the histological status of the pleural disease at that time remains unknown. In patients with suspected malignant pleural effusion, additional sampling methods, including pleural brushing combined with cell-block preparation, may provide complementary diagnostic material when conventional pleural fluid cytology is nondiagnostic (28). In this patient, the subsequent lymph-node biopsy confirmed squamous histology and enabled molecular comparison with the original tumor.

There is currently no established standard treatment for EGFR-mutant lung adenocarcinoma after squamous transformation. Carboplatin plus nab-paclitaxel was selected on the basis of the newly confirmed squamous phenotype. Tislelizumab was added after multidisciplinary discussion as an individualized treatment decision, given the patient’s previous treatment exposure and the lack of an established treatment for this setting. This regimen was extrapolated from treatment strategies for advanced squamous NSCLC rather than from evidence specific to EGFR-mutant tumors after transformation. PD-L1 status was unavailable at treatment initiation and subsequently proved negative, with a TPS of <1% and a CPS of <1. Because EGFR-mutant NSCLC generally derives limited benefit from immune checkpoint inhibitor monotherapy, and because the patient received combination therapy, the contribution of tislelizumab to the observed response cannot be determined. Chemotherapy alone would also have been a reasonable option (29–31). Treatment-related adverse events were limited to a grade 1 decrease in white blood cell count and grade 1 peripheral sensory neuropathy, with no documented immune-related adverse events during the reported follow-up period.

This report has several limitations, including its single-patient design, relatively short post-transformation follow-up, lack of pleural sampling at the first suspected progression, and use of different sequencing panels for the paired specimens. The absence of pleural tissue meant that the timing of transformation could not be determined. Differences in panel coverage, tumor-cell content, and other pre-analytical and analytical characteristics also prevented direct comparison of variant allele frequencies. More comprehensive genomic, transcriptomic, and epigenetic analyses would be required to clarify the evolutionary relationship between the lesions. Tumor mutation burden and immune-cell infiltration were not evaluated, and CK5/6, GATA3, and p16/human papillomavirus testing could not be performed because of limited biopsy material. Thus, although the findings favored acquired squamous transformation, an occult second primary squamous cell carcinoma cannot be completely excluded.

This case illustrates squamous histological transformation as a rare resistance pattern in EGFR-mutant lung adenocarcinoma after long-term osimertinib treatment. It also highlights the value of repeat tissue biopsy and integrated pathological and molecular reassessment when progression follows an atypical pattern. Further studies are needed to clarify the underlying mechanisms and define optimal treatment strategies after squamous transformation.

## Data Availability

The original contributions presented in the study are included in the article/**Supplementary Material**. Further inquiries can be directed to the corresponding authors.

## Supplementary material

Supplementary Figure 1 Histopathological and immunohistochemical findings in metastatic pulmonary adenocarcinoma involving the station 6 and station 13 lymph nodes and a pleural nodule. **(A–D)** Station 6 lymph node. Hematoxylin and eosin (H&E) staining shows metastatic adenocarcinoma within the lymph node **(A)**. The tumor cells show positive nuclear staining for TTF-1 **(B)**, positive granular cytoplasmic staining for Napsin A **(C)**, and negative staining for p40 **(D)**. **(E–H)** Station 13 lymph node. H&E staining shows metastatic adenocarcinoma **(E)**. The tumor cells are positive for TTF-1 **(F)** and Napsin A **(G)** and negative for p40 **(H)**. **(I–K)** Pleural metastatic nodule. H&E staining shows adenocarcinoma involving the pleura **(I)**, with positive staining for TTF-1 **(J)** and Napsin A **(K)**. **(L, M)** Additional low-power views of serial sections from the station 6 lymph node, showing the metastatic focus on H&E staining **(L)** and positive TTF-1 immunostaining **(M)**. Because the metastatic focus was small and the panels were obtained from different serial sections, its position and configuration differ between panels **(L, M)**. Original magnification: ×200 for panels **(A–K)** and ×40 for panels **(L, M)**. Scale bars: 100 μm for panels **(A–K)** and 500 μm for panels **(L, M)**.
